# Everyday Water-Related Emergencies: A Didactic Course Expanding Wilderness Medicine Education

**DOI:** 10.21980/J8WS90

**Published:** 2023-07-31

**Authors:** Geoffrey B Comp, Erica Burmood, Molly Enenbach, Savannah Seigneur

**Affiliations:** * Creighton University School of Medicine-Phoenix, Valleywise Health Medical Center, University of Arizona College of Medicine Phoenix, Department of Emergency Medicine, Phoenix, AZ

## Abstract

**Audience:**

This small group session is appropriate for any level of emergency medicine resident physicians.

**Introduction:**

Drowning is defined as the process of experiencing respiratory impairment from submersion or immersion in liquid. It is the third leading cause of unintentional injury-related deaths worldwide, accounting for 7% of all injury-related deaths.^1^ Our group sought to improve resident education regarding the basics of water safety and rescues as an event developed by our wilderness medicine (WM) interest group. With the growing number of WM Fellowships, specialty tracks, interest clubs and the regular inclusion of WM topics in residency didactics, exposure to WM topics has increased greatly.^2^ There is a large overlap between wilderness medicine and the field of emergency medicine. Both require stabilization, improvisation, and the treatment of environmental/exposure illnesses. It is imperative that emergency medicine physicians understand the complex pathophysiology of drowning, as well as recognize and manage potential associated traumatic injuries including fractures and critical hemorrhage. Our goal is to provide additional curricular instruction on prehospital management of water-related emergencies and related injuries to emergency medicine residents.

**Educational Objectives:**

By the end of the session, the learner will be able to: 1) describe the pathophysiology of drowning and shallow water drowning, 2) prevent water emergencies by listing water preparations and precautions to take prior to engaging in activities in and around water, 3) recognize a person at risk of drowning and determine the next best course of action, 4) demonstrate three different methods for in-water c-spine stabilization in the case of a possible cervical injury, 5) evaluate and treat a patient after submersion injury, 6) appropriately place a tourniquet for hemorrhage control, and 7) apply a splint to immobilize skeletal injury.

**Educational Methods:**

A group of 16 resident learners received a thirty-minute introduction discussion (with open discussion) regarding water safety, basic water rescue methods, and submersion injury pathophysiology. They then progressed through three stations designed to emphasize select skills and knowledge related to submersion injury management, water rescue, and tourniquet and splint placement.

**Research Methods:**

Participants completed a six-item questionnaire after the event designed to help gage participant comfort level of treatment, management, and experience regarding water safety, drowning, and related traumatic emergencies. Each item was ranked from 0 for “strongly disagree” to 10 for “strongly agree.” Total mean scores before and after were compared.

**Results:**

Sixteen individuals participated in the sessions and survey. The total mean score for the six-item analysis increased following the workshop (26.3 before versus 46.9 after, p = 0.001). The positive improvement in all categories indicated increased comfort in the topics of the small group sessions, with the largest improvement in the question about comfort in effectively evaluating and treating a patient presenting to the ED after a submersion injury.

**Discussion:**

Utilizing discussions and hands-on group sessions increased residents’ perceived learning. This model can be applied to an extensive number of wilderness medicine topics for learners of all levels. For individuals with time-restrictive schedules, this model is an efficient mode of learning and teaching drowning and injury management skills with the potential for further topics and future courses.

**Topics:**

Wilderness medicine, water safety, pathophysiology of drowning, in-water rescues, in-water cervical spine stabilization, management of drowning in the ED, splinting, tourniquets.

## USER GUIDE

[Table t3-jetem-8-3-sg1]

**Table t3-jetem-8-3-sg1:** 

List of Resources:	
Abstract	1
User Guide	3
Appendix A: Introductory Discussion Outline	8
Appendix B: Prehospital and Hospital Submersion Injury Management Discussion Outline	11
Appendix C: Active Water Rescue Discussion Outline	14
Appendix D: Splinting/Tourniquet Discussion Outline	17

**Learner Audience:**

Medical Students, Interns, Junior Residents, Senior Residents

**Time Required for Implementation:**

Two hours (30 minutes of introduction followed by three 30-minute stations)

**Recommended Number of Learners per Instructor**:

4–6

**Topics:**

Wilderness medicine, water safety, pathophysiology of drowning, in-water rescues, in-water cervical spine stabilization, management of drowning in the ED, splinting, tourniquets.

**Objectives:**

By the end of the session the learner will be able to:

1. Describe the pathophysiology of drowning and shallow water drowning
2. Prevent water emergencies by listing water preparations and precautions to take prior to engaging in activities in and around water
3. Recognize a person at risk of drowning and determine the next best course of action
4. Demonstrate three different methods for in-water c-spine stabilization in the case of a possible cervical injury
5. Evaluate and treat a patient after submersion injury
6. Appropriately place a tourniquet for hemorrhage control
7. Apply a splint to immobilize skeletal injury

### Linked objectives and methods

The goal of this small group session was to provide both a fun and informative educational session focused on drowning including prevention, management, and water rescue. The management of additional injuries associated with watersports outside of drowning, including the assessment and management of skeletal and soft tissue injuries and c-spine management, were also incorporated into the session. The full group of learners received a thirty-minute introduction discussion regarding water safety, water rescue methods, and submersion injury pathophysiology (objectives one, two, and five). They then progressed through three stations designed to emphasize select skills and knowledge related to submersion injury management (objective five), water rescue (objective three and four), as well as tourniquet and splint placement (objectives six and seven). The selection of both small group and small station format were utilized to highlight multiple educational learning theories and facilitate progressive teaching responsibility as well as to provide enough time for both education and skills practice.

### Recommended pre-reading for instructor

- Auerbach P, Constance B, Freer L. Trauma Emergencies: Assessment and Stabilization. *Field Guide to Wilderness Medicine*. 5^th^ ed. Elsevier; Philadelphia, PA; 2019:119–141.
- Auerbach P, Constance B, Freer L. Orthopedic Injuries, Splints, and Slings. *Field Guide to Wilderness Medicine*. 5^th^ ed. Elsevier; Philadelphia, PA; 2019:168–227.
- Auerbach P, Constance B, Freer L. Drowning and Cold-Water Immersion. *Field Guide to Wilderness Medicine*. 5^th^ ed. Elsevier; Philadelphia, PA; 2019:641–648.

### Small Group Stations

After a 30-minute group introduction discussion, our 16 learners were broken into three groups (five–six learners each) for further demonstration and practice. These three groups were: prehospital and hospital submersion injury management, in-water safety and rescue, and tourniquet and splint placement. Because this event was held outside, PowerPoint or other digital presentation supplements were not used or created. Instead, the educators gave a brief oral presentation followed by a hands-on component. A poster board was used for the initial discussion and a photo is included as an example. Outlines for all the stations are included as supplementary documents.[Fig f1-jetem-8-3-sg1][Fig f2-jetem-8-3-sg1]

**Figure f1-jetem-8-3-sg1:**
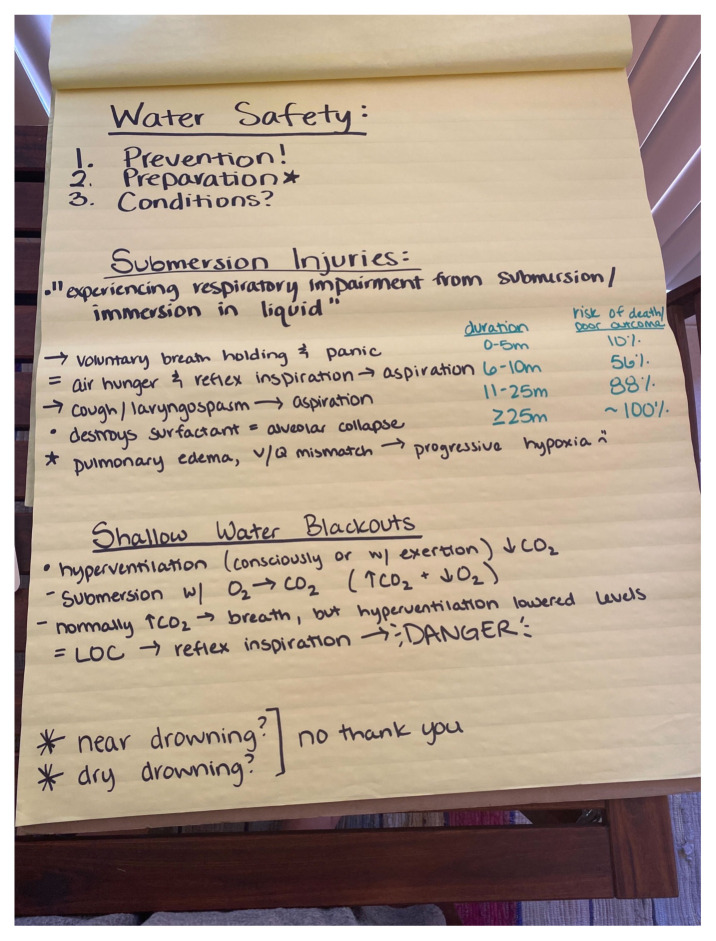


**Figure f2-jetem-8-3-sg1:**
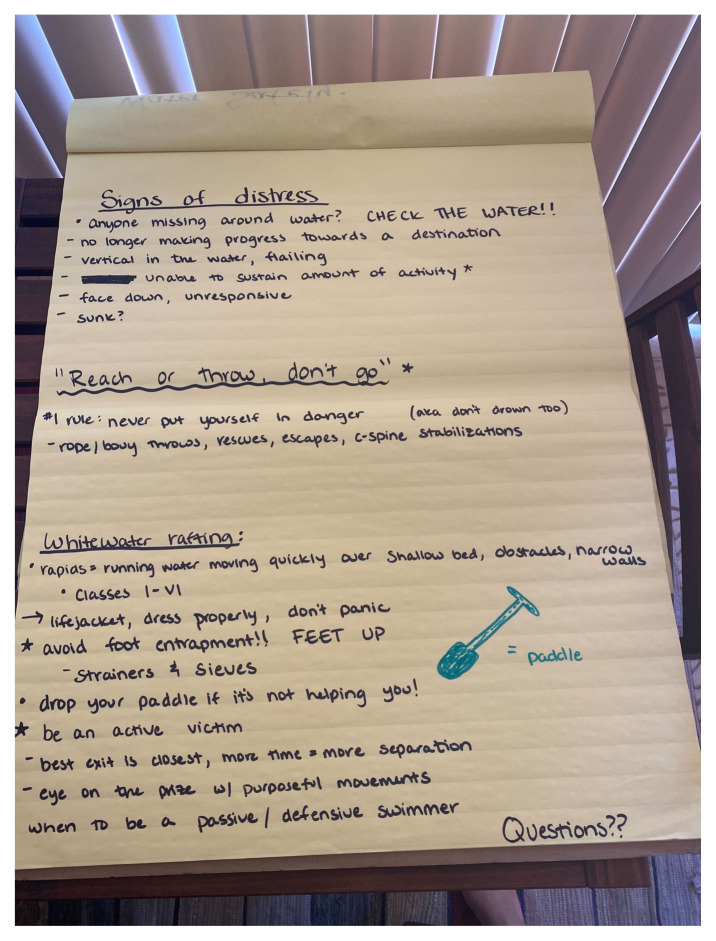


### Materials

Many of the materials used for the course were the personal property of the attending and the department of wilderness medicine. The resident educators should be familiar with the location of the exercise and understand the materials they will be using or demonstrating. Estimated prices are included next to the materials in the individual sections.

### Results and tips for successful implementation

The course is best implemented in an outdoor environment with residents with an interest in wilderness medicine. The event took place at Saguaro Lake outside of Phoenix, Arizona on July 9, 2022. The event was held at Butcher Jones Cove, where the group had access to fresh water and desert landscape, allowing for the demonstration and practice of skills as described in the learning objectives. Sixteen learners of varying skill levels participated in the event. The event was reviewed and approved by the institutional IRB prior to implementation.

We created a retrospective one-sample, pre- and post-test educational evaluation consisting of six questions. Participants self-reported their confidence and understanding of various topics (Table 1). The six-question form asked participants to describe their level of agreement with each of the six statements on a ten-point scale with 0 for “Strongly Disagree” to 10 for “Strongly Agree.” There was one additional question asking for any additional comments or opportunities for improvement.

The total mean scores for the six items before and after the course were analyzed using the t-test. Of the 16 learners who participated in the course, 16 (100%) completed the questionnaire. For the primary analysis, the total mean score of the six-question survey increased following the workshop (26.3 before versus 46.9 after, p = 0.001). Question 1 had the most significant change in pre-test to post-test confidence according to the McNemar’s Test, with a pre-test level of agreement (rank 6–10) of 1 with post-test level of agreement (rank 6–10) of 15, p = 0.0002. Of the 16 participants, 31% completed the Post Workshop Comments (see Table 2).

Of those who completed the post-workshop comments, one individual marked N/A with 50% of the remaining individuals stating no suggestions for change and the other 50% with suggested changes. In future courses, we will implement additional time for skills practice. A potential barrier to the exact replication of this educational activity is if there is no access to a large body of water to practice specific water skills. However, many of the same ideas can be taught through demonstrations on dry land, or by replacing specific sessions with either additional time for skills practice or expanding on the initial topics. Depending on the individual, institution, and location, other topics including hypo/hyperthermia, altitude sickness, avalanche rescue, and improvised carries can be tailored to the interested audience and adjusted for audience skill level.

### Associated content/sections of didactics

Appendix A: Introductory Discussion Outline

Appendix B: Prehospital and Hospital Submersion Injury Management Discussion Outline

Appendix C: Active Water Rescue Discussion Outline

Appendix D: Splinting/Tourniquet Discussion Outline

### Pearls

Drowning/submersion injury definitions

- Describe the pathophysiology of drowning and shallow water drowning.^1^,^4^
  ○ Submersion injury: “The process of experiencing respiratory impairment from submersion/immersion in liquid.”
  ○ Voluntary breath-holding -> panic -> air hunger and reflex inspiration -> aspiration, cough, laryngospasm -> further aspiration -> destruction of surfactant, alveolar collapse -> pulmonary edema, ventilation-perfusion (V/Q) mismatch -> hypoxia
  ○ Shallow water blackouts: hyperventilation (consciously/unconsciously) -> decreased CO2 -> underwater O2 converted to more CO2 -> initially decreased CO2 levels do not trigger swimming to the surface -> level of consciousness (LOC) with reflex inspiration
  ○ The terms “dry drowning” and “secondary drowning” were made popular in the media, especially during 2018–21. During this time there were multiple reports of a child aspirating and eventually developing complications days/weeks later. As unfortunate as these cases were, the terms are not specific and are incorrect regarding the pathophysiology of submersion injuries. The outcomes were more likely secondary to other disease processes.

Water safety, signs of distress, and in-water rescue techniques:

- Prevent water emergencies by listing specific preparations and precautions to take prior to engaging in activities in and around water. 5
  ○ Prevent emergencies by preparation. Research your water conditions prior to entering, and consider water temperature, depth, current, rocks, boats, visibility, flora/fauna, etc.
  ○ Be sure to wear a life jacket if you are uncomfortable in the water and have extra units available in case of emergency.
- Recognize a person at risk of drowning and determine the next best course of action. 6
  ○ Check the water first when someone is missing. A rapid response and rescue provides a better chance to prevent a submersion injury.
  ○ Signs of distress: No longer making progress towards a destination, vertical in the water, unable to sustain the amount of energy they are expending, face down and unresponsive.
  ○ Never put yourself in danger while attempting to rescue another swimmer; it is better to utilize objects around you that could assist in rescues such as ropes, poles, or flotation devices. We suggest the practice: “reach or throw, don’t go.”

Associated Injury Management

- Demonstrate three different methods for in-water cervical spine stabilization in the case of a possible cervical injury.^7^
  ○ Only stabilize the c-spine if a victim has had a traumatic event.
    ■ Streamline hold: rescuee’s arms overhead in streamlined position holding over biceps (preferred method and easiest in deep water).
    ■ One hand posterior on the head with the forearm extending down the swimmer’s back with your other hand anterior on the chin extending down the swimmer’s chest. The swimmer’s arms will be at their side.
    ■ Hands grasping shoulders with head held tightly between your forearms (least favorite but good in shallow water).

Drowning/submersion injury treatment

- Evaluate and treat a patient after submersion injury.^8^
  ○ Reversing hypoxia is the key component of drowning management. Make sure to optimize oxygenation and ventilation.
    ■ Obtain accurate vitals and provide supplemental oxygen as indicated. Options include nasal cannula, non-rebreather, non-invasive positive pressure ventilation, bag-valve-mask, or intubation depending on your patient’s mental status and ability to participate.
    ■ Assess if your patient would benefit from a jaw thrust maneuver to further airway patency and elevate the head to prevent further aspiration.
    ■ As with any patient, be sure to allow for adequate exhalation to avoid significant hypercapnia.
  ○ Evaluate for the potential for other injuries or environmental issues guided by history and physical exam.
    ■ In an unresponsive or critical patient, advanced trauma life support (ATLS) protocols should be observed to systematically evaluate airway, breathing, circulation, exposure, and disability.
    ■ By also performing a thorough secondary survey, you will be less likely to miss associated head trauma, cervical injuries, bodily wounds, or abnormal vital signs (hypotension, hypothermia, bradycardia).
  ○ In the ED or hospital, systematically resuscitate the patient with a focus on the correction of acidosis, hypoxemia, and hypoperfusion, as well as close investigation for cardiac and pulmonary emergencies.

Injury Management

- Appropriately place a tourniquet for hemorrhage control.^9^
  ○ Perform a physical exam to assess for circulation and signs of profuse or pulsatile bleeding.
  ○ First, apply direct pressure to control bleeding. Next, apply a hemostatic dressing if available. If still bleeding, apply a tourniquet.
  ○ A tourniquet is a device that is applied directly to skin, to stop all distal blood flow. Tourniquets are commercially available or can be improvised from various materials.
  ○ Combat Application Tourniquet (CAT) Demonstration
    ■ Place the tourniquet proximal to, but as close as possible to the bleeding site. Tighten as much as possible and secure. Tighten further by twisting the windlass, then secure by tucking it under the plastic clip. Write the time of tourniquet placement on the outside of the strap.
  ○ Windlass Demo
    ■ Ideally, use wide (3–4 in) and flat material made from clothing, towels, etc. Never use wire, rope, or other thin material because it would likely cut and damage the skin.
- Demonstrate how and when to apply a splint to immobilize skeletal injury.
  ○ Physical Exam to Assess Injury and Neurovascular status^10^
    ■ Palpate along long bones proximally to distally, palpating for deformity and crepitus. If crepitus is present without deformity, place a splint.
    ■ Assess the patient’s active range of motion. If unable to perform, gently assess the passive range of motion. If the joint has swelling or resistance to motion, place a splint.
    ■ If the joint is dislocated, perform reduction after completing a neurovascular assessment. Place the splint carefully to prevent the recurrence of dislocation.
    ■ To assess neurovascular status, assess the distal pulses, color, temperature, capillary refill, and sensation of the extremity.
    ■ Perform serial neurovascular examinations of the extremity to ensure proper splint placement and to assess injury status.
  ○ If there is an open fracture present, and help is greater than eight hours, use *clean* water to irrigate the wound. Cover with a clean compression dressing. Treat with broad-spectrum antibiotics if able because open fractures pose a risk for osteomyelitis.
  ○ SAM splint Demo:
    ■ A SAM Splint is a thin sheet of aluminum in between two layers of closed-cell foam. When U-shaped bending is created along the long axis of the splint, it becomes rigid.
    ■ SAM splints can be used to immobilize any long bone or be improvised into a cervical collar.
    ■ Triangular Bandage: For upper extremity splints, the provider can fold up the bottom half of the shirt and pin to the top to utilize the shirt as a sling.

## Figures and Tables

**Table 1:** Water Safety/Living in the Desert Workshop Questionnaire.

Items Evaluated Pre- and Post-Workshop
I can effectively evaluate and treat a patient presenting to the ED after a submersion injury
I understand the pathophysiology of drowning
I am familiar with the basics of water safety and issues related to aquatic rescue
I have experience with water rescue techniques
I am comfortable placing a tourniquet for hemorrhage control
I am able to place a splint to effectively immobilize a potential skeletal injury

**Table 2:** Post Workshop Comments

Would be fun to do more water rescue simulations.
This course was great! The practice with water rescue techniques was awesome. I also liked how we talked about reduction techniques as well.
More activities involving actual water rescue techniques. We didn’t get a lot of practice so it’s hard to say that my comfort level is significantly improved with these techniques.
No changes, was a great experience.

## SMALL GROUPS LEARNING MATERIALS

### Appendix A Introductory Discussion Outline

1. Discussion of the day
  - Why are we here?
    - Discussion about water safety drowning and related injuries
  - Learning objectives
    - Describe the pathophysiology of drowning and shallow water drowning
    - Prevent water emergencies by listing water preparations and precautions to take prior to engaging in activities in and around water
    - Recognize a person at risk of drowning and determine the next best course of action
    - Demonstrate three different methods for in-water c-spine stabilization in the case of a possible cervical injury
    - Evaluate and treat a patient after submersion injury
    - Appropriately place a tourniquet for hemorrhage control
    - Apply a splint to immobilize skeletal injury
  - Outline movement through the day
    - 30 minute group introduction discussion
    - Then three group break out for further demonstration and practice
      1. Prehospital and hospital submersion injury management
      2. In-water safety and rescue
      3. Tourniquet and splint placement
2. Drowning
  - A process resulting in primary respiratory impairment from submersion or immersion in a liquid medium. The victim may live or die during and after this process. The outcomes are classified as death, morbidity, and no morbidity.
    - airway goes below the surface of the liquid (submersion)
    - water splashes over the face (immersion)
  - These phrases should be avoided: “near drowning,” “dry or wet drowning,” “secondary drowning,” “active and passive drowning,” and “delayed onset of respiratory distress.”
3. Epidemiology
  - Drowning is the third leading cause of unintentional injury death worldwide, accounting for 7% of all injury-related deaths.
  - In 2019, there were an estimated 236,000 annual drowning deaths worldwide.
  - 4,000 fatal unintentional drownings
    - 11 drowning deaths per day.
  - 8,000 nonfatal drownings
    - 22 nonfatal drownings per day.
  - More children ages one to four die from drowning than any other cause of death.
  - For children ages 5–14, drowning is the second leading cause of unintentional injury death after motor vehicle crashes.
  - 64% of all victims are under age 30
  - 26% of all victims are under age 5
4. Pathophysiology
  - Respiratory failure resulting in hypoxemia, cardiac ischemia, cardiac arrest, and neurologic injury
  - In survivors, the long-term morbidity reflects the severity and duration of cerebral anoxia experienced
  - The drowning process begins when the victim’s airway lies below the surface of a liquid medium (usually water)
  - voluntary breath-holding
  - Small amounts of water are aspirated from the oropharynx/larynx; water is aspirated into the airway
    - 1 to 3 mL/kg
  - coughing occurs as a reflex response
  - laryngospasm may occur (rapidly terminated by the onset of brain hypoxia)
  - continued aspiration -> hypoxemia -> loss of consciousness and apnea
  - Final mode of death involves cardiac dysrhythmia: tachycardia -> bradycardia -> pulseless electrical activity (PEA) -> asystole
5. Water safety
  - Prevent emergencies
  - Wear a life jacket if you are uncomfortable in the water or could be knocked unconscious (rafting)
  - Know your limits
  - Appropriate supervision
  - Prepare: enter the water, get a breath, swim a short distance, exit the water
  - Never swim alone, call for help
  - Swim sober
6. Know your water conditions:
  - Temperature
  - Rip currents
  - Depth
  - Visibility
  - Flora/fauna
7. Signs of distress:
  - If a child is missing around water -> CHECK THE WATER FIRST, seconds count
  - No longer making progress towards a destination
  - Vertical in the water, unable to sustain amount of activity
  - Face down, unresponsive

### Appendix B Prehospital and Hospital Submersion Injury Management Discussion Outline

**Materials:**

- None

**Small Groups:**

- Small groups to discuss the topic of prehospital and hospital submersion injury management.

**Discussion questions:**

- What is the primary goal of treatment for all drowning injuries?
  ○ Remove/evacuate the patient, reverse hypoxia, and assist with ventilation.
- What is the main goal of laboratory evaluation in an obtunded drowning patient?
  ○ Assistance with investigating other causes of altered mental state in the differential diagnosis.
  ○ For patients whose mental status fails to respond to resuscitation or in whom the initial cause of submersion is unknown, laboratory testing for causes of altered mental status should be considered.

**Educational Outline:**

1. Initial Management
  - Resuscitation
    - Airway/breathing prioritization
      1. Establishing an airway and providing oxygen are priorities in the initial resuscitation of a drowning patient.
      2. Most important aspect of treatment is to reverse cerebral hypoxia by providing oxygen to the brain by whatever means available.
      3. Drowning can produce a gasping pattern or apnea while the heart is still beating, and the person may need only ventilation.
      4. The European Resuscitation Council recommends five initial rescue breaths instead of two (as recommended by the American Heart Association) because the initial ventilations can be less effective in drowning victims.
    - ACLS
      1. Victims should be taken as quickly as possible to dry land where effective CPR can be initiated.
      2. Reversal of hypoxia with ventilations and compressions should not be delayed by applying an automated external defibrillator (AED).
        - Really only if the possibility of ventricular fibrillation (VF) is the cause of drowning.
  - Trauma/Spinal immobilization
    - Spinal immobilization should be considered in patients with evidence of spinal injury, such as focal neurological deficit or history of high-risk activity, and in patients who exhibit altered mental status.
    - Retrospective studies of drowning patients found the incidence of cervical spine injuries was low (0.5%–5%) and that most such injuries were related to diving from a height.
  - Water Removal?
    - Heimlich not recommended.
2. In the ED
  - Initial evaluation
    - History
      1. Description of scene, time submerged, location, potential known contaminants, water temperature, water contamination, vomiting, type of rescue, precipitating events.
    - Exam
      1. Dyspnea
      2. Air hunger due to the reduction in both ventilation and perfusion.
      3. Irritant receptors in the airways will be stimulated by the aspirated water, causing the patient to cough.
      4. Blockage of the airways and fluid in the lungs will result in rales, rhonchi, and wheezing.
    - Vital signs are vital
      1. Pulse oximetry, accurate temperature.
    - Differential
      1. Primary drowning? Drowning as a result of other medical emergencies?
        - For patients whose mental status fails to respond to resuscitation or in whom the initial cause of submersion is unknown, laboratory testing for causes of altered mental status should be considered.
  - Continued Management
    - Fix the hypoxia and assist with ventilation
      1. Administer oxygen if hypoxic.
      2. Noninvasive positive pressure ventilation (NIPPV) if patient not altered.
      3. The treatment of persons who have been rescued from drowning resembles that of patients with acute respiratory distress syndrome (ARDS).
  - Labs
    - Measurements of electrolytes, blood urea nitrogen, creatinine, and hematocrit are rarely helpful; abnormalities are unusual.
    - Correction of electrolyte imbalance is rarely needed.
    - Laboratory evaluation to investigate possible concomitant emergencies.
    - Metabolic acidosis occurs in many patients.
      1. Rehydration
    - Venous blood gas (VBG) may be indicated to guide respiratory interventions.
  - ECG
  - Radiology
    - CXR/CT.
    - Other imaging based on differential.
  - Antibiotics
    - There is no evidence to support empiric antibiotic therapy in the treatment of drowning patients.
  - Steroids
    - Not routinely administered.

### Appendix C Active Water Rescue Discussion Outline

**Materials:**

- Paddles: ~$30
- Rescue buoy: ~$40
- Water “noodle”: ~$3
- Life jacket:

**Location:**

Anywhere located by water deep enough to submerge yourself with a stable out of water area nearby. This could be a lake with shoreline, swimming pool with deck, deep water with boat or floating platform in the water. Assure that learners can be both in and out of the water. It is helpful to have both shallow and deeper areas of water to practice the rescues in various environments; however, one depth will be adequate. This can also be simulated or addressed through active discussion if a body of water is not accessible.

**Discussion questions:**

- Why do we practice “reach or throw, don’t go!” in regard to in-water rescue?
  ○ This is the safest way to aid a distressed swimmer by avoiding putting yourself in additional danger. By entering the water yourself you run the risk of becoming a dressed swimmer and adding another person who requires rescue.
- Name two signs of a distressed swimmer who may require additional assistance/rescue:
  ○ Swimmers are no longer making forward progress in the water towards their destination.
  ○ Vertical in the water (often flailing) and are unable to move productively or tread water.
  ○ Unable to sustain the amount of energy output they are using.
  ○ Passive victims are often face down in the water without movement.
  ○ Sunk/sinking in the water.

**Small group:**

- During the active water portion, learners were grouped into pairs
- Skills were demonstrated by the instructor with one learner volunteer
- Individuals then practiced the skill with their partner
- Learners switched places to practice again
- After each activity the group reconvened and questions were answered
- Assure that any learner not comfortable in the water has a life jacket readily available while on land and appropriately worn while in the water

**Educational Outline:**

1. “Reach or throw, don’t go”:
  - First demonstrate with a learner how to perform various out of water rescues by reaching and/or throwing various items near a distressed swimmer
    - Paddles, buoys, water noodles, additional life jackets can all be used.
    - Always attempt to throw the item to the rescuees without hitting their head.
  - Emphasize to never place yourself in danger when performing a rescue.
  - Reach assist: reach with your arm, leg, or an object (noodle, paddle) to assist a swimmer.
  - Throw assist: throw a flotation device near a swimmer to provide buoyancy.
    - Can be a buoy with a rope (to then pull the swimmer to safety) or a life jacket to provide flotation until rescue can occur.
  - Practice: start with one learner on land and one in the water, attempt out of water rescues with multiple items.
    - Learners switch places.
2. In-water rescues:
  - Both learners in the water for this portion, preferable deep water.
  - Always approach a distressed swimmer from behind, yell as approaching to announce yourself.
  - Never let them face you and grab onto your neck; this will sink both of you and is the most dangerous.
    - If this occurs, duck underwater (the distressed swimmer will reactively let go of you).
    - Then swim away.
    - Last resort: unfortunately one way to get someone to let go of you is to punch them in the stomach. Please don’t practice this on your partners but in a life or death situation it may save your life.
  - Once you are near your swimmer, you can place any floatation device you have (buoy, noodle, life jacket) between you and the swimmer.
    - Alternatively, you can hand the swimmers the floatation device if you think this will allow them adequate support while being able to swim yourself to safety.
  - Wrap your arms under their arms from behind, and let their head fall to one of your shoulders.
    - Do not get hit in the head; this is much easier with a unconscious swimmer.
  - Lay back and kick both of you to safety while providing reassuring information to the person being rescued.
  - Once at safety, allow the swimmer to climb out or be lifted out first.
3. Streamline hold:
  - Everyone in shallow water to start,; swimmer in streamline position (hands over head with hands over each other).
  - Rescuer holds pressure over swimmer’s biceps to provide inline c-spine stabilization
  - Switch and allow other learner to practice.
  - What if your distressed swimmers were on their stomach? How would you roll someone over while maintaining c-spine stabilization?
    - Demonstrate going underwater to roll your rescuee to their back smoothly while maintaining inline c-spine stabilization.
  - Practice in deep water as well; likely the easiest way to maintain stabilization while egg-beater kicking with your feet to keep both yourself and your rescuee afloat.
4. Double arm hold:
  - Demonstrate in shallow water with one hand posterior on the head (on the occiput) with the forearm extending down the swimmer’s back, and with your other hand anterior on the chin, extending down the swimmer’s chest.
  - Requires shallow water to provide adequate stabilization and support.
5. Shoulder hold:
  - Demonstrate in shallow water standing superior to the patient with hands on shoulders squeezing their head gently between your forearms.
    - Imagine holding c-spine precautions in the trauma bay.
  - Also requires shallow water to provide adequate stabilization and support.

### Appendix D Splinting/Tourniquet Discussion Outline

**List of materials:**

- SAM splint (or another malleable splint)
- CAT tourniquet (Combat Application Tourniquet)
- SWAT-Tourniquet (Stretch, Wrap, and Tuck)
- Triangle bandage

**Location:**

- Splinting can be taught in an indoor or outdoor setting provided there is adequate room for people to sit and lay on the ground.
- For each injury, upper or lower extremities may be used to practice a variety of splinting locations.

**Small Groups:**

- Skills were initially demonstrated by the instructor on a learner.
- After the initial demonstration, individuals practiced each skill on a partner and the instructor was available for individual instruction.
- Once each partner in the group was able to practice each skill, the small group convened and questions were answered.
- For each learning objective, the above steps were completed.

**Discussion Questions:**

- Do you need to immobilize the cervical spine of every submersion victim? Whose do you need to immobilize?
  ○ No, only those with a history or signs of traumatic injury. Routine c-spine immobilization is not warranted based on history of submersion alone.
  ○ “Submersion victims are at risk for C-spine injury only if they have also sustained a traumatic injury. Routine C-spine immobilization does not appear to be warranted solely on the basis of a history of submersion.”
    ■ Watson RS, Cummings P, Quan L, Bratton S, Weiss NS. Cervical spine injuries among submersion victims. *J Trauma*. 2001 Oct;51(4):658–62. PMID: 11586155. At: doi: 10.1097/00005373-200110000-00006
- When applying a tourniquet, where is the best placement for the device on the extremity?
  ○ Apply tourniquet immediately proximal to the bleeding wound, as close to the wound as possible.
  ○ This is to stop the bleeding while minimizing the amount of tissue affected by decreased blood flow distal to the tourniquet.

**Educational Outline:**

1. Injury Management
  - Appropriately place a tourniquet for hemorrhage control.
    - Perform a physical exam to assess for circulation and signs of profuse or pulsatile bleeding.
    - First, apply direct pressure to control bleeding. Next, apply a hemostatic dressing if available. If still bleeding, apply a tourniquet.
    - A tourniquet is a device that is applied directly to skin to stop all distal blood flow. Tourniquets are commercially available or can be improvised from various materials.
    - Combat Application Tourniquet (CAT) Demonstration
      1. Place the tourniquet proximal to, but as close as possible to the bleeding site. Tighten as much as possible and secure. Tighten further by twisting the windlass, then secure by tucking it under the plastic clip. Write the time of tourniquet placement on the outside of the strap.
    - Windlass Demo
      1. Ideally, use wide (3–4 in) and flat material made from clothing, towels, etc. Never use wire, rope, or other thin material because it would likely cut and damage the skin.
  - Demonstrate how and when to apply a splint to immobilize skeletal injury.
    - Physical Exam to Assess Injury and Neurovascular status.
      1. Palpate along long bones proximally to distally, palpating for deformity and crepitus. If crepitus is present without deformity, place a splint.
      2. Assess the patient’s active range of motion. If unable to perform, gently assess the passive range of motion. If the joint has swelling or resistance to motion, place a splint.
      3. If the joint is dislocated, perform reduction after completing neurovascular assessment. Place the splint carefully to prevent recurrence of dislocation.
      4. To assess neurovascular status, assess the distal pulses, color, temperature, capillary refill, and sensation of the extremity.
      5. Perform serial neurovascular examinations of the extremity to ensure proper splint placement and to assess injury status.
    - If there is an open fracture present, and help is greater than eight hours, use *clean* water to irrigate the wound. Cover with a clean compression dressing. Treat with broad spectrum antibiotics if able because open fracture is at risk for osteomyelitis.
    - SAM splint Demo:
      1. Sam Splint is a thin sheet of aluminum in between two layers of closed cell foam. When U-shaped bending is created along the long axis of the splint, it becomes rigid.
      2. Sam splint can be used to immobilize any long bone or be improvised into a cervical collar.
      3. Triangular Bandage: For upper extremity splints, the provider can fold up the bottom half of the shirt and pin to the top to utilize the shirt as a sling.
